# Very High Density of CHO Cells in Perfusion by ATF or TFF in WAVE Bioreactor™. Part I. Effect of the Cell Density on the Process

**DOI:** 10.1002/btpr.1704

**Published:** 2013-05-21

**Authors:** Marie-Françoise Clincke, Carin Mölleryd, Ye Zhang, Eva Lindskog, Kieron Walsh, Véronique Chotteau

**Affiliations:** School of Biotechnology, Cell Technology Group, KTH (Royal Institute of Technology)SE-106 91, Stockholm, Sweden; GE Healthcare Bio-Sciences ABBjörkgatan 30, SE-75184, Uppsala, Sweden; GE Healthcare Bio-Sciences CorpWestborough, MA, 01581

**Keywords:** wave bioreactor, alternating tangential flow hollow fiber, tangential flow filtration hollow fiber, perfusion, cell broth viscosity, Chinese Hamster Ovary cell

## Abstract

High cell density perfusion process of antibody producing CHO cells was developed in disposable WAVE Bioreactor™ using external hollow fiber filter as cell separation device. Both “classical” tangential flow filtration (TFF) and alternating tangential flow system (ATF) equipment were used and compared. Consistency of both TFF- and ATF-based cultures was shown at 20–35 × 10^6^ cells/mL density stabilized by cell bleeds. To minimize the nutrients deprivation and by-product accumulation, a perfusion rate correlated to the cell density was applied. The cells were maintained by cell bleeds at density 0.9–1.3 × 10^8^ cells/mL in growing state and at high viability for more than 2 weeks. Finally, with the present settings, maximal cell densities of 2.14 × 10^8^ cells/mL, achieved for the first time in a wave-induced bioreactor, and 1.32 × 10^8^ cells/mL were reached using TFF and ATF systems, respectively. Using TFF, the cell density was limited by the membrane capacity for the encountered high viscosity and by the pCO_2_ level. Using ATF, the cell density was limited by the vacuum capacity failing to pull the highly viscous fluid. Thus, the TFF system allowed reaching higher cell densities. The TFF inlet pressure was highly correlated to the viscosity leading to the development of a model of this pressure, which is a useful tool for hollow fiber design of TFF and ATF. At very high cell density, the viscosity introduced physical limitations. This led us to recommend cell densities under 1.46 × 10^8^ cell/mL based on the analysis of the theoretical distance between the cells for the present cell line. © 2013 American Institute of Chemical Engineers *Biotechnol. Prog*., 29:754–767, 2013

## Introduction

Although batch and fed-batch processes are the most widely used production systems for mammalian cells, perfusion processes are gaining interest.^1^ Perfusion mode provides a constant environment favorable to the cells by continuous by-product removal and nutrient addition.^2^–^5^ In comparison to batch and fed-batch modes, the perfusion mode allows prolonging healthy cultures, potentially at high cell density, as well as a short residence time of the product in the bioreactor. This is more favorable for the product quality and is mandatory for the production of unstable glycoprotein, for example, factor VIII.^6^ Another advantage of the perfusion mode is the use of smaller bioreactors compared with fed-batch processes, which implies benefits such as reduced clean-in-place operation and the possibility to use disposable bioreactors instead of stainless steel reactors due to the smaller working volumes. Besides its use in biopharmaceutical production bioreactor, perfusion mode can also be used for high cell density seed bioreactors, cell bank manufacturing, or the production of proteins as research tool. In this latter case, the production of the product of interest at high cell density can advantageously compensate for a low cell specific productivity. This requires only some process tuning, possibly using medium renewal in excess compared to the cell demand and therefore saving labor for process development. Perfusion processes have also drawbacks: more challenging processes from technical and sterility point of views, large harvest volumes continuously accumulating and necessitating further processing, large medium volumes, and generation of multiple harvest batches per culture run.

Several methods for cell separation in perfusion are available based on different physical principles: (i) filtration by tangential flow or cross-flow filter, for example, hollow fiber filter (HF), vortex-flow filter, spin-filter, perfusion floating filter; (ii) acceleration-based settlers: inclined settler, acoustic settler, centrifuge, hydrocylone.^7^–^10^ The separation device robustness is critical for efficient operation and achievement of high cell densities.^11^ Disposable bioreactors equipped with disposable cell separation device, which are robust and easy to exchange in case of fouling or malfunction, can offer a solution alleviating technical and sterility challenges occurring during perfusion. Today a few technologies are available as disposable devices: centrifuge Centritech, tangential flow filtration (TFF), alternating tangential flow (ATF, Refine Technology), perfusion floating filter,^4^ hydrocyclone,^9^ CellTank.^12^ This study focussed on TFF and ATF as these technologies were the only ones among those named above, reported to support high cell density, having moderate equipment cost, allowing renewal of the device during the culture, and compatible with wave-induced bioreactor (see below). Contrary to conventional filtration with cell broth moved in flow direction orthogonal to the filter surface, in TFF the cell suspension is flowing tangentially to the membrane potentially preventing membrane fouling.^11^,^13^–^15^ The ATF uses TFF but with the cell broth flow direction alternated using a diaphragm pump, creating less shear stress than the TFF peristaltic pump, and with a cycle time around 1 min.^16^–^18^ The alternated movement creates a back flush in the filter membrane, which can help preventing fouling.^19^

The wave-induced bioreactor has gained increasing interest since its description by Singh in 1999 and its commercialization.^20^ Today other types of disposable bioreactors are available with homogenization by stirring, shaking, or obtained by the recirculation flow in reactors where the cells are immobilized. The demand for these technologies for biopharmaceutical production has increased this last decade due to their short turnover, a reduction of cleaning and sterilization steps, implying reduction of contamination risk and cleaning validation.^21^,^22^ Wave-induced bioreactors are nowadays used for the manufacturing of protein or specialized cells and widely used for cell expansion steps. These bioreactors are easy to operate, the oxygenation is performed in absence of gas sparging, and therefore foaming is significantly reduced. The deleterious effect of sparging has been reported to be a potential limitation in the achievement of high cell density in perfusion mode by Mercille et al.^3^ even though very high cell densities of Per C6 cells have been reported in nondisclosed perfused stirred tank processes.^23^ According to Singh,^20^ a good nutrient distribution and an excellent oxygen transfer from the headspace without damaging shear of gas bubbles are achieved in wave-induced bioreactor. Furthermore, compared to oxygenation by gas transfer from the headspace, oxygenation by sparging is technically more challenging in small scale due to the foam formation. In other words, a wave-induced bioreactor designed for high cell density perfusion combined with adequate cell separation device can potentially be an excellent tool for applications like high cell density expansion for seed train, cell bank manufacturing, or protein production.

A CHO cell line expressing a monoclonal antibody IgG1 was used as model for this investigation, aiming at studying and comparing high cell density perfusion processes in a wave-induced bioreactor either with ATF or TFF. WAVE Bioreactor™ system 20/50 with 10 L disposable Cellbag™ customized with two dip tubes was connected either to ATF or TFF mounted with ReadyToProcess™ HF. A preliminary characterization of the systems was performed in water model. Then perfusion runs were performed in which ATF and TFF systems were first characterized at moderate cell density (∼25 × 10^6^ cells/mL), then at higher cell density (∼10^8^ cells/mL) focussing on the influence of the operating parameters on the cell growth and viability. This study was followed by an evaluation of the cell density limits. This article is focusing on developing the perfusion processes and studying the effect of the cell density. The applications of these perfusion processes, MAb production and cell bank manufacturing, are presented in Part II.^24^

## Materials and Methods

### Perfusion culture processes

A research cell line CHO DHFR^−^ producing IgG1 was used as model for the study. The cells were thawed and expanded in Ex-Cell 302 medium with 4 mM glutamine and 100 nM methotrexate (all SAFC), in shake flask (37°C, 5% CO_2_, 100 rpm). The cells were inoculated from pooled shake flask cultures into the bioreactor. In the bioreactor, the medium was animal component-free IS CHO CD XP with hydrolysate, supplemented with 3% IS-CHO Feed-CD XP, 4 mM glutamine (all Irvine Scientific), and 0.1 mg/mL streptomycin/100 mL/U penicillin G/0.25 *μ*g/mL amphotericin B (all SAFC). An alternative medium mix was shortly tested on days 11–18 in TFF#6 run and days 1–6 in ATF#8 run: PFCHO Liquid Soy medium (Thermo Fisher) supplemented with 3% CHO Feed Bioreactor Supplement (SAFC). Supplementations of glucose and glutamine were performed according to the cell need. Up to 50 ppm antifoam C (AF) (Dow Corning) was added by boost or by intermittent pumping in the bioreactor when foam occurred.

The bioreactor was a 10 L Cellbag™ mounted on WAVE Bioreactor™ controlled by a WAVEPOD™ Integrated Controller (all GE Healthcare). The Cellbag™ was mounted with two dip tubes: for TFF both dip tubes were connected to the HF via a recirculation pump (Watson Marlow 620S pump with two-roller pump head) and for ATF one of the dip tubes was connected to a HF mounted on an ATF-2 device (Refine Technology). For both systems, the tube internal diameter (ID) was 3/8" for the recirculation loop and 1/2" for the part of the dip tube inside the Cellbag™. The pump tube of the TFF recirculation loop had 1/2" ID. The cells were continuously circulated through the lumen side of an external vertically oriented HF, entering at the HF lower end for the TFF and upper end for ATF systems. In both systems, the HF was a ReadyToProcess™ filter (RTPCFP-2-E-4X2MS, GE Healthcare) with 0.2 *μ*m pore size, 1 mm lumen, 60 cm nominal flow path length, 850 cm^2^ filter area, and 50 polysulfone fibers and ReadyMate™ disposable aseptic connectors were used (HFs were water rinsed for 30 min, allowed to stand in water overnight, and autoclaved prior to use). The pressure regulating valve, pressure rising flow, exhaust flow, and orifice were automatically set by the ATF regulator connected to a vacuum pump Ilmvac membrane pump MP 105 Z. The pressures in the recirculation and the harvest lines were monitored with on-line pressure sensors SciPres (piezoresistive sensors by SciLog BioProcessing Systems) placed in the tubes and sterilized by autoclaving.

The perfusion run set points (SP) of dissolved oxygen concentration (DO), pH, temperature, and working volume were 35%, 7.0, 37°C, and 4 L. The DO was controlled by rocking rate variation with rocking rates 20–28 rpm and angles 6°–8°, see Table 2. Manually tuned oxygen addition of 20–100% was performed continuously into the headspace. The aeration rate was manually tuned between 0.025 and 0.15 vvm. The pH was controlled by adding 0.5 M Na_2_CO_3_ or CO_2_ into the headspace. The runs were initiated in batch mode, and the perfusion was started at cell density range 2–3 × 10^6^ cells/mL. The medium additions were automatically pumped, controlled by the WAVEPOD™ to maintain the working volume at 4±0.3 kg, measured by a load cell. The harvest was continuously withdrawn on the permeate side of the HF by a peristaltic pump (Alitea) manually adjusted at least twice a day to tune the perfusion, 1–10 reactor volume/day (RV/day). Except in run TFF#10 after day 35, the cell bleeds were performed manually based on the cell counting by discarding a part of the cell broth and replacing it by fresh medium, after which a new cell counting was performed. In run TFF#10 after day 35, the cell bleed was performed by manually tuned continuous pumping of the cell broth. Samples were taken once or twice a day from the bioreactor and the permeate line. The cell density and the viability were measured by Bioprofile FLEX (Nova Biomedical) after dilution with phosphate buffered saline solution to obtain a cell density <10^7^ cells/mL—two measurements of the cell densities≥2 × 10^8^ cells/mL were performed using two different dilutions. pH, partial pressure of CO_2_ (*p*CO_2_), cell diameter, concentrations of glucose, lactate, glutamine, and ammonia were measured by Bioprofile FLEX (Nova Biomedical). The viability was also determined by lactate dehydrogenase activity (LDH) using enzymatic kit (Promega) and calculated as (total cell density−perfusion rate × dead cell density by LDH)/total cell density. The cell broth viscosity was measured by a DV-II+ Viscometer (Brookfield Engineering Labs) in cell broth concentrated at different cell densities and cultivated in shake flasks.

### Cell specific rate and shear rate calculations

The cell specific rates of apparent cell growth (*µ*), glucose (*q*_glc_) and glutamine (*q*_gln_) consumption and lactate (*q*_lac_) and ammonium (*q*_amm_) production were calculated as:


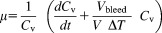
(1)


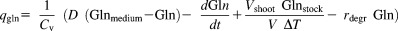
(2)


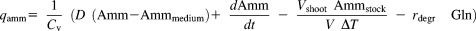
(3)

with *V*, working volume; *D*, perfusion rate*; C*_v_, viable cell density; Gln, glutamine concentration; Amm, ammonium concentration in the culture; *V*_bleed_, bleed volume; *S*_stock_, substrate stock solution concentration used for the shoot added at interval Δ*T =*1 day; *V*_shoot_, substrate shot volume; *S*_medium_, substrate concentration in fresh medium. *r*_degr_ is the degradation rate of glutamine, measured to be 0.046 day^−1^ in IS CHO CD XP medium at 37°C. Similar equations were used for *q*_glc_ and *q*_lac_ without degradation term.

The cell specific perfusion rate (CSPR) was defined as


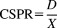
(4)

The shear rate *γ* was calculated as


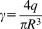
(5)

where *q* and *R* are the flow rate in the fiber lumen and the lumen radius.

## Results and Discussion

### Development of the perfusion process set-up

**Figure 1:**
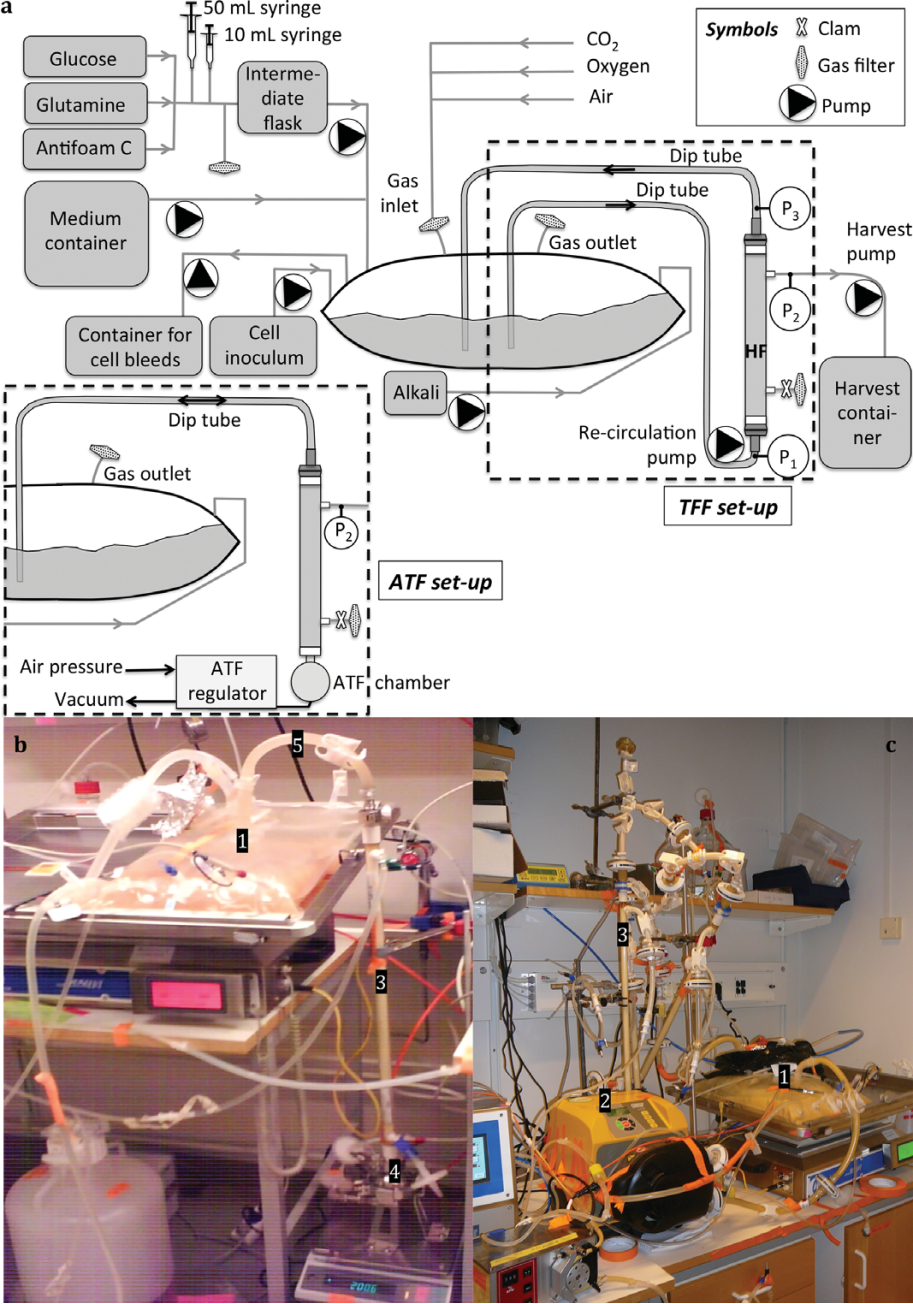
(a) Process set-up for TFF- and ATF-based perfusion; *P*1, *P*2, and *P*3 are the HF pressure indicators at HF inlet, permeate, and retentate, respectively; (b) and (c) photos of the ATF- and TFF systems in the lab at KTH: Bag with culture (1), recirculation pump (2), HF (3), ATF (4), and dip tube (5). The (c) photo was taken at cultivation day 44 with a cell density ≥2 × 10^8^ cells/mL. The TFF system was mounted with three HFs as unused HFs were left connected however clamed.

**Figure 2:**
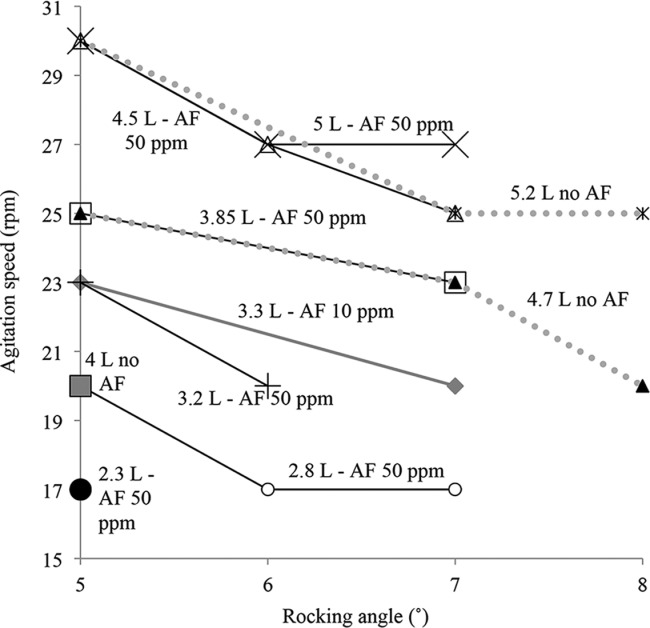
Occurrence of air in the dip tube (between ATF and bioreactor) as a function of the rocking angle, the rocking rate, and the working volume in the absence (no AF) or presence of antifoam (10 or 50 ppm) in 1 g/L pluronic water solution. In the graph, the data points are the limits where no air occurrence was observed while at higher rocking angle or higher rocking rate or lower volume, air occurrence was observed.

The bioreactor was either connected to an ATF or a TFF system (see set-up in Figure 1a). Pictures of the systems in operation at KTH's lab are given in Figures 1b,c. The Cellbag™ was connected to the HF of the ATF or the TFF via two dip tubes piercing the upper surface of the bag (in the vicinity of the center) and anchored on the bag bottom at ports with large opening allowing cell broth circulation. The dip tube length was as short as possible for optimal operation of the ATF system and prevention of bubble occurrence in the dip tube. Bubbles in the dip tube were susceptible to impair the filtration function and cause shear damage to the cells. The air occurrence was preliminary studied using a water model in the ATF system. We identified that at working volume ≥4.5 L in presence of 50 ppm AF no air accumulated in the dip tube even at high rocking rates and angles, see Figure 2. AF presence partially prevented the air occurrence however not completely even though no foam was formed on the liquid surface.

### Consistency and Reproducibility of Perfusion Cultures

**Figure 3:**
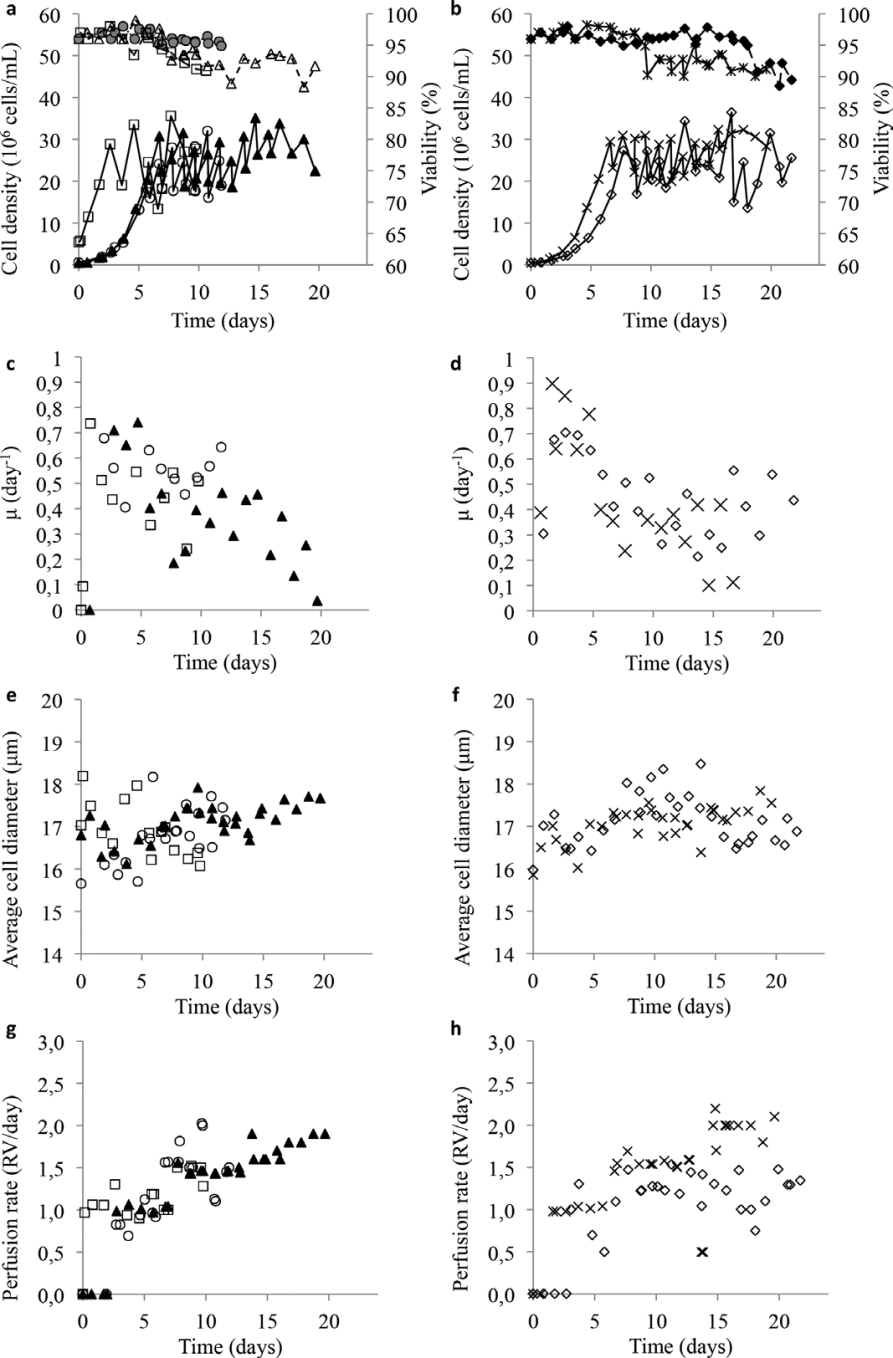
Perfusion processes using ATF (left), runs ATF#5 (circle), ATF#8 (square), ATF#9 (triangle), or TFF (right), runs TFF#6 (diamond), TFF#10 (cross), stabilized around 20–30 × 10^6^ cells/mL by daily cell bleeds: (a, b) viable cell density (continuous), viability (dotted), (c, d) growth rate, (e, f) average cell diameter, and (g, h) perfusion rate. The cultures were inoculated at cell density 0.4–0.5 × 10^6^ cells/mL except in ATF#8, which was inoculated from TFF#6 at 5 × 10^6^ cells/mL cell density.

Two perfusion processes were developed using either ATF or TFF as cell separation device. In a first part of the study, the process was developed, the settings were tested, and the reproducibility and consistency at ∼30 × 10^6^ cells/mL cell density were assessed in three ATF runs and two TFF runs. In all the runs except ATF#8, the bioreactor was inoculated at 0.44–0.52 × 10^6^ cells/mL, and the perfusion was started at 1 RV/day on day 2 or 3 with a cell density range 2–3 × 10^6^ cells/mL. ATF#8 was inoculated at 5.5 × 10^6^ cells/mL with cells taken from TFF#6 run cell bleed at day 11, and the perfusion (1 RV/day) was initiated immediately. For both retention devices, the cell growth was exponential with a growth rate 0.55 day^−1^, and a cell density of 20 × 10^6^ cells/mL was reached with 1 RV/day perfusion rate at day 6, see Figure 3. An exception was run TTF#6, in which the growth was slower the first 3 days, probably due to the pH, which was accidentally ≤6.8. The cells were maintained between 20 and 35 × 10^6^ cells/mL for 3–16 days by performing manual daily cell bleeds with a perfusion rate ∼1.5 RV/day, see Figure 3 and Table 1. The cell viability was high during the cultures, mostly above 90%, and the cell diameter (*D*_cell_) was 17.1 *μ*m in average. The cells grew with an average specific growth rate of 0.35 day^−1^ in both ATF and TFF systems. During these cultivations, the *p*CO_2_ varied between 2 and 13 kPa. Table 1 lists parameters of all the ATF and TFF runs.

**Table 1:** Perfusion Parameters for all the Experiments

Process	ATF					TFF		
		
Cell density	20–35 × 10^6^ cells/mL			≥ 35 × 10^6^ cells/mL		20–35 × 10^6^ cells/mL		≥ 35 × 10^6^ cells/mL
				
Run	ATF#5	ATF#8	ATF#9	ATF#15A	ATF#15B	TFF#6	TFF#10	TFF#10
Culture duration (day)	12.7	10.6	21.7*	12.8	9.7	21.7	19.6	47.7
Duration of stable cell density (day)	6.5	8	16	–	–	14	14	17.1†
Max viable cell density (10^6^ cells/mL)	32	36	35	132	123	36	31	214
Max total cell density (10^6^ cells/mL)	33	39	38	137	125	38	34	224
Average perfusion rate (RV/day)	1.4	1.2	1.5	3.1	2.6	1.2	1.5	3.9
Maximal perfusion rate (RV/day)	2	1.5	1.9	6	6.1	1.5	2.2	10
Total spent medium (L)	56	55	129	129	100	99	110	717
Average μ (day^−1^)	0.54	0.45	0.36	0.33‡	0.31‡	0.39	0.42	0.28‡; 0.26§; 0.15††
*p*CO_2_ (kPa)	4–13.6	1.8–7.1	2.6–17	2.4–21.9	3.4–22.2	2.7–14.3	3.1–16.9	3.1–31.2
Average of CSPR	0.07	0.055	0.06	0.05	0.05	0.06	0.06	0.055
Recirculation flow rate (L/min)	1	1	0.7; 1 from day 9	1	1	0.3	0.7; 1 from day 8	1
Shear rate (1/s)	3,400	3,400	2,400; 3,400 from day 9	3,400	3,400	1,000	2,400; 3,400 from day 8	3,400

* Total duration was 27 days with the last 5 days devoted to parameter testing.

† At *C*_v_ ≍ 0.85–1.3 × 10^8^ cells/mL.

‡ At *C*_v_≥35 × 10^6^.

§ At *C*_v_ ≍ 10^8^.

†† At *C*_v_ ≥1.5 × 10^8^ cells/mL.

**Table 2:** Rocking Rates and Oxygenation Parameters used for ATF and TFF Systems During the Perfusion Cultures

	Rocking Rate (rpm)		Rocking Angle (°)		O_2_ Supply (%)		Gas Flow (L/min)	
				
Viable Cell Density (10^6^ cells/mL)	ATF	TFF	ATF	TFF	ATF	TFF	ATF	TFF
∼0–5	20		6		20–30		0.1	
∼5–35	22–26		6–7		20–50		0.1–0.2	
∼35–60	22–26		7	8	50–75		0.2	
∼60–80	22–26	24–26	7	8	75–80		0.2	
∼80–130	22–26	24–26	7–8	8	100	90–95	0.2–0.6	0.2–0.3
∼130–160	–	24–26	–	8	–	80	–	0.3
∼160–200	–	24–26	–	8	–	80	–	0.3–0.6
∼200–214	–	25–28	–	8	–	100	–	0.3–0.5

### CSPR-based feed control

A key factor in perfusion is the rate of medium renewal, that is, the perfusion rate. We choose to correlate linearly the perfusion rate with the cell density to avoid nutrients' limitations, to prevent toxic by-product accumulation, and to provide a constant environment to the cells. In other words, a CSPR was applied as introduced by Ozturk^25^ where CSPR is the volume of medium renewed per cell and day, see Materials and Methods section. The principle was that if a CSPR providing satisfying cell environment was identified for a moderate cell density, the same CSPR should be applied for very high cell densities. This fit also well our focus, which was to study process development and not medium optimization. A CSPR of 0.05 nL/cell/day was selected based on previous work.^26^ Notice that we choose to supply glucose and glutamine according to the cell need and not correlated to medium renewal to reduce the medium consumption. During the practical experimental work, the CSPR varied mostly between 0.05 and 0.06 nL/cell/day. In run ATF#5, it was observed that increasing the CSPR up to 0.08 nL/cell/day did not improve the cell growth or the maximal cell density leading to the conclusion that 0.05–0.06 nL/cell/day CSPR was sufficient in this process. According to Konstantinov et al.,^27^ depending on the process, CSPR may vary in the range 0.05–0.5 nL/cell/day, and 0.05 nL/cell/day is considered to be very low when operating at high cell densities in the range of 40–80 × 10^6^ cells/mL.

### Very high CHO cell densities

**Figure 4:**
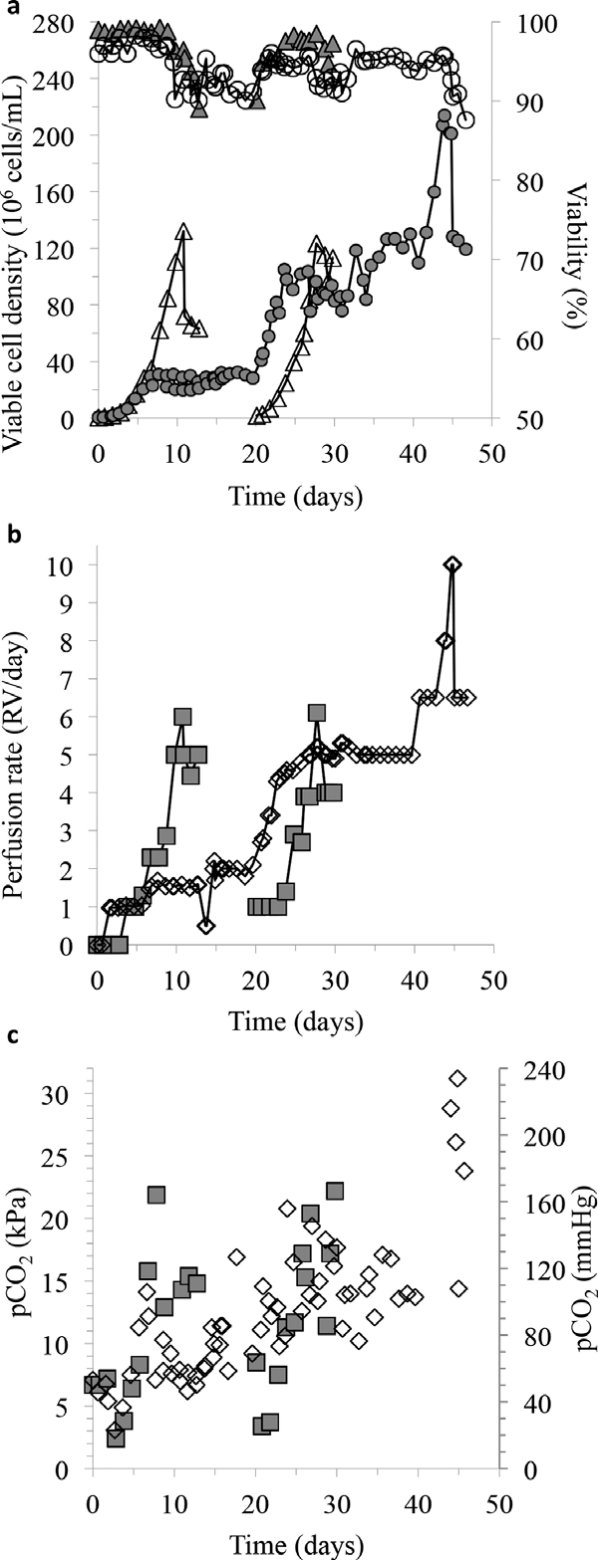
Assessment of the perfusion systems using ATF, run ATF#15A-B (triangle), or TFF, run TFF#10 (circle): (a) cell density and viability; (b) perfusion rate in ATF#15A-B (square) and TFF#10 (diamond); (c) *p*CO2 same symbols as (b); a secondary *y*-axis is represented in mmHg units for the reader easiness. Maximal cell densities of 2.14 and 1.32 × 10^8^ cells/mL were achieved using TFF and ATF, respectively. The cell density was maintained at 10^8^ cells/mL by daily bleeds for 2 weeks in TFF#10.

Runs TFF#10 and ATF#15A-B were performed to identify the maximal cell densities achievable in these systems by increasing the perfusion rate according to a constant CSPR. As can be seen in Figure 4, in experiment ATF#15A, a cell density of 1.32 × 10^8^ cells/mL was reached after 10 days of exponential growth with a final perfusion rate of 6 RV/day. Reaching this density coincided with spontaneous interruption of ATF alternated flow, potentially due to insufficient vacuum action to pull the highly viscous cell broth from the bioreactor into the HF. The HF was replaced and a batch culture (with sustained ATF motion without medium removal) was carried out between days 13 and 19, data not shown. The perfusion was resumed on day 20. The run was named ATF#15B, performed from day 20 to the end. During ATF#15B run, a maximal cell density of 1.23 × 10^8^ cells/mL was reached, again with spontaneous interruption of ATF alternated flow, confirming the first observed maximal cell density. At this stage, the bioreactor was artificially pressurized at 0.02–0.03 bar and after 2 min, the ATF alternated flow resumed, showing that a higher effect than delivered by the vacuum in place could restore the ATF function. A comparable effect would also have been obtained in case of a nondisposable pressurized bioreactor: the bioreactor pressure adding up to the vacuum in the ATF chamber would have helped the flow motion during the vacuum cycle, which is the most challenging effect to obtain among pressure and vacuum. A cell bleed was then performed to reduce the cell density around 10^8^ cells/mL, and the culture was continued without changing the HF. Normal ATF function was observed at 10^8^ cells/mL cell density maintained by cell bleeds during 2 days, before termination due to time constraint. This showed that the HF function was not altered and that fouling was not the reason for stopped ATF function. It showed that healthy high cell density culture could be obtained somewhat around 10^8^ cells/mL with this process set-up.

Using the TFF in run TFF#10, exponential cell growth at similar rate as with ATF was observed between 0.28 and 1.05 × 10^8^ cells/mL from day 20 to 24. The cell density was then maintained in growing stage between 0.9 and 1.3 × 10^8^ cells/mL by daily cell bleeds during more than 2 weeks showing the consistency of this process for this cell density range, see Figure 4. Finally, the maximal cell density was tested by increasing the perfusion rate accordingly with the cell density. The cell density reached 2.14 × 10^8^ cells/mL on day 44 but the cells stopped then to grow. A cell density ≥2 × 10^8^ cells/mL was observed during 2 days with 8 and 10 RV/day perfusion rate. At this stage two limitations occurred: a high pressure of 1 bar in the recirculation loop caused by the viscosity of the very high cell density and a too high value of pCO_2_, 31 kPa, see Figure 4c. Furthermore, the oxygenation began to be limiting as well.

**Figure 5:**
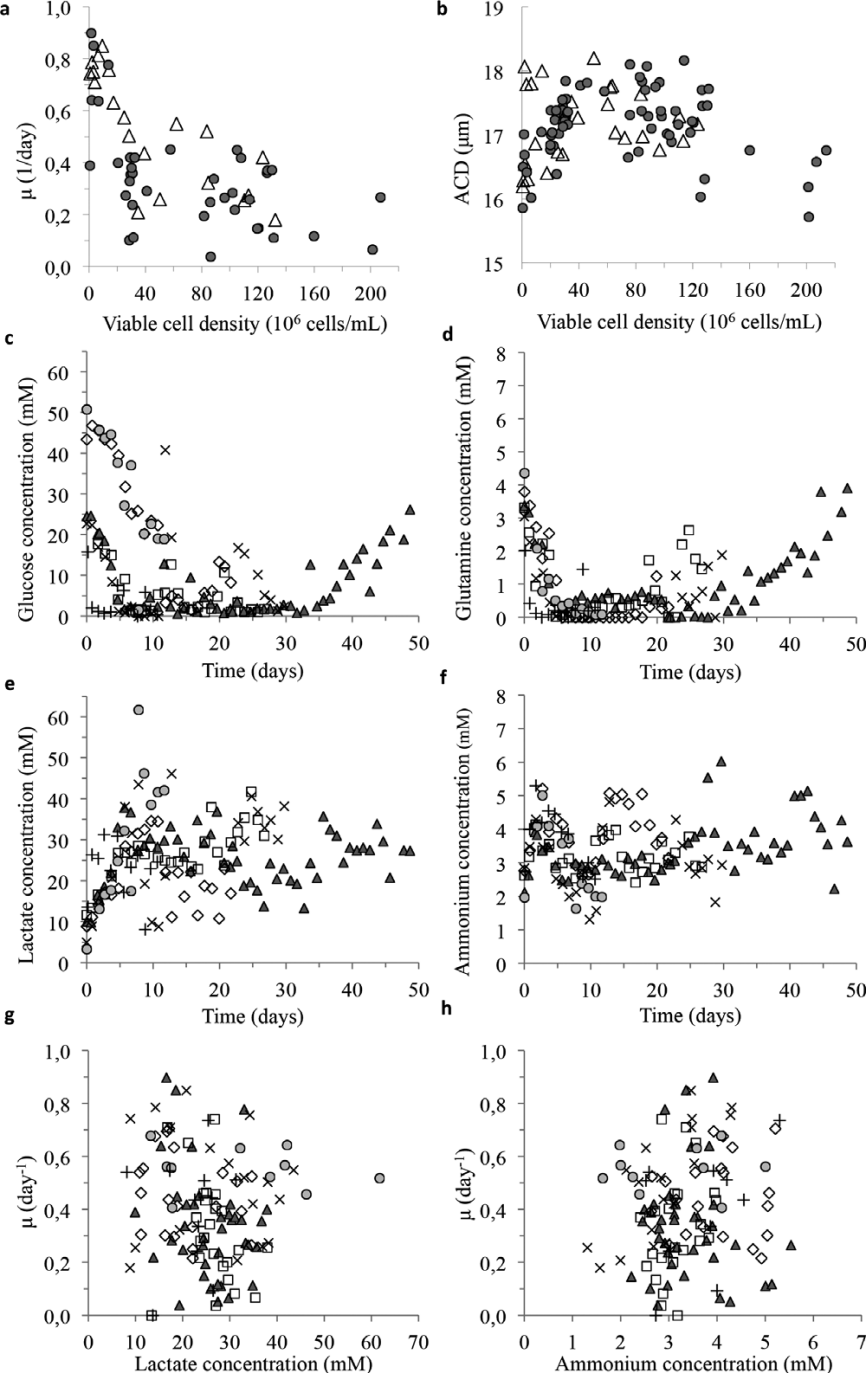
(a) Growth rate and (b) average cell diameter in ATF#15 (circle) and TFF#10 (triangle) runs. The growth rate was highest at density ≤ 20 × 106 cells/mL, comparable or somewhat lower at 0.8–1.2 × 108 cells/mL than at 20–40 × 106 cells/mL and lower above 1.6 × 108 cells/mL. Concentrations of glucose (c), glutamine (d), lactate (e), and ammonium (f) during runs ATF#5 (circle), ATF#8 (+), ATF#9 (square), ATF#15A-ATF#15B (×), TFF#6 (diamond), TFF#10 (triangle). Growth rate in function of lactate (g) or ammonium (h) concentrations.

During both ATF and TFF runs, the cells were cultivated at high and very high cell densities with a cell viability maintained above 90% (mostly around 95%), see Figure 4a. This high viability measured by Trypan blue exclusion by BioProfile FLEX was confirmed by LDH, data not shown. The growth rate variation with the cell density is given in Figure 5a and Table 1. After a faster growth at cell density ≤0.2 × 10^8^ cells/mL, the growth rate stayed constant or slightly decreased from ∼0.4 day^−1^ to ∼0.3 day^−1^ between 0.2 and 1.3 × 10^8^ cells/mL cell density. Above 1.5 × 10^8^ cells/mL, the growth rate decreased to ∼0.15 day^−1^. The cellular MAb production was comparable in both systems but this production was partly retained by the HF and in a more severe way using the TFF, see Part II.^24^

The cell diameter, given in Figure 5b, was comparable using ATF or TFF between 0.3 × 10^8^ and 1.3 × 10^8^ cells/mL with average 17.3 µm measured by BioProfile FLEX—notice that this average was not altered by the presence of cell aggregates since the cells were always single cells for this cell line. Above 1.5 × 10^8^ cells/mL, it decreased to 16.4 µm.

As can be observed in Figure 4c, the *p*CO_2_ pattern was somewhat correlated to the cell density. *p*CO_2_ stayed below 22 kPa most of the time but reached 31 kPa at 2 × 10^8^ cells/mL density. 31 kPa, or 232 mm Hg, is a level known to affect the growth rate.^28^–^30^

Achievement of a cell density up to 50 × 10^6^ cells/mL is reported in the literature using perfusion based on hollow fiber device.^5^Kyung et al.^31^ have shown that high cell density of 10^8^ HEK293 cells/mL in suspension could be achieved using an internal hollow fiber module with cellulose ester membrane. Adams et al.^32^ obtained a cell density of 1.5 × 10^8^ Per C6 cells/mL in a wave-induced bag with an internal perfusion membrane after 8 days of cultivation followed shortly by membrane blockage. Wang et al.^33^ achieved a cell density of 1.04 × 10^8^ *Drosophila schneider* 2 cells/mL, maintained during 6 days, also using a wave-induced perfusion system equipped with an internal membrane. It is the first time that data of cell densities of 0.9–1.3 × 10^8^ cells/mL maintained during 18 days are presented. Although used in industry, ATF-based perfusion and concentrated fed-batch processes are seldom published^5^,^17^ and are not disclosed even if very high Per C6 cell densities have been announced.^23^ To our knowledge, it is the first time that CHO cell density above 10^8^ cells/mL is published in a disclosed ATF process.

### Cell metabolism and medium cell metabolism and medium effect

**Figure 6:**
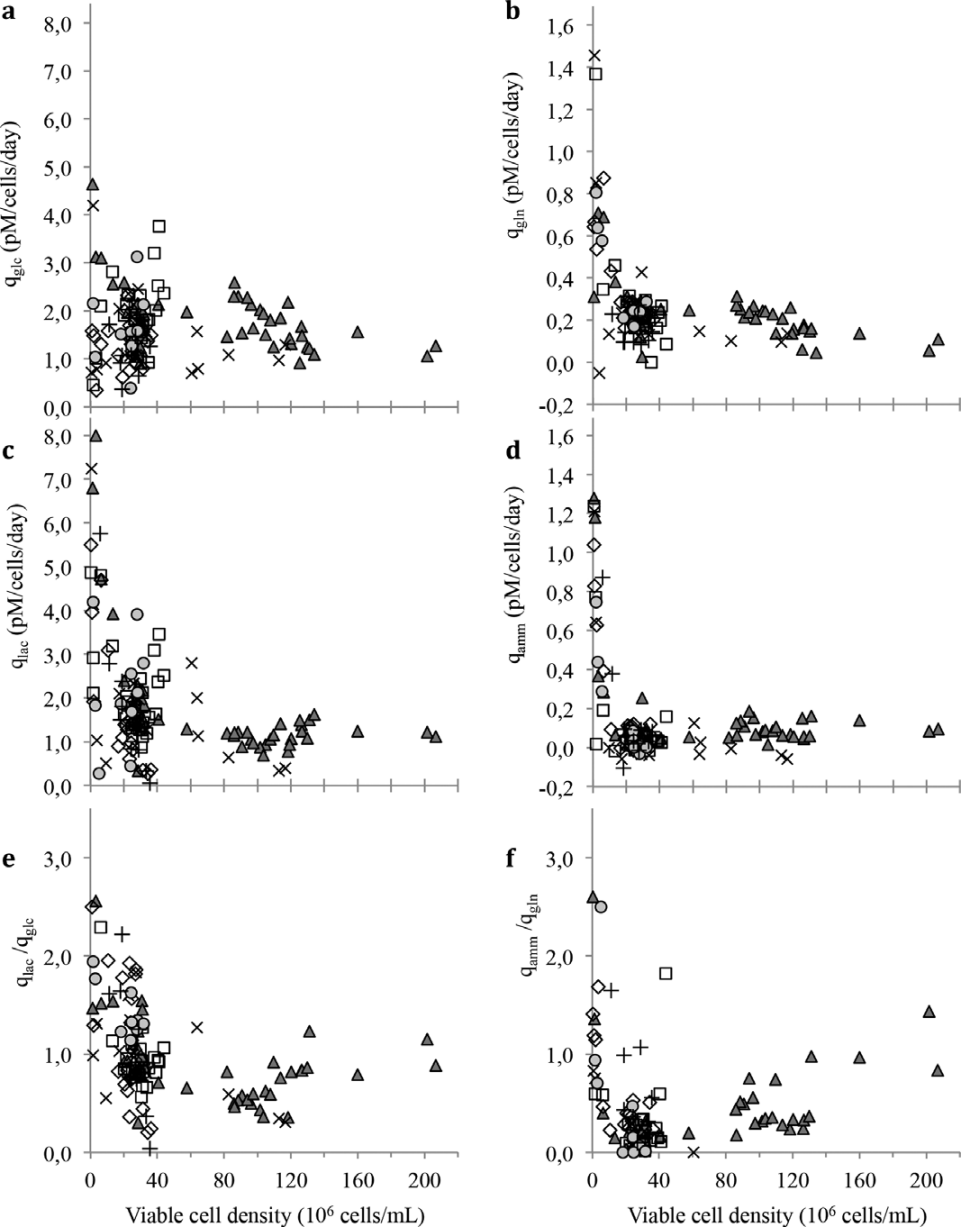
Cell specific rates: (a) *q*_glc_, (b) *q*_gln_, (c) *q*_lac_, (d) q_amm_ in function of the viable cell density during runs ATF#5 (circle), ATF#8 (+), ATF#9 (square), ATF#15A-ATF#15B (×), TFF#6 (diamond), TFF#10 (triangle). Metabolic ratios: (e) *q*_lac_/*q*_glc_; (f) *q*_amm_/*q*_gln_.

The concentrations of glucose, glutamine, lactate, and ammonium in the bioreactor are given in Figures 5c–f. Except for the first days in all the runs and the last days of TFF#10, the residual concentrations of glucose and glutamine were often very low (≤6 and ≤0.7 mM, respectively, sometimes measured as ∼0); however, these energy sources were present in the fresh medium and supplementary added as well. The lactate and ammonium concentrations were mostly ≤46 and ≤6 mM, respectively. The lactate range was comparable to the range observed in fed-batch runs while the ammonia concentration reached up to 18 mM in fed-batch (see Part II^24^). The cell specific rates of consumption and production of these metabolites are represented as a function of the cell density in Figures 6a–d. The *q*_gln_, *q*_lac_, and *q*_amm_ were significantly higher at cell density ≤10^7^ cells/mL compared to larger densities showing a higher metabolism associated with a higher growth rate. *q*_glc_, *q*_gln_, *q*_lac_, and *q*_amm_ were comparable at cell densities between 10^7^ and 2 × 10^8^ cells/mL except for *q*_gln_, which decreased slightly above 1.6 × 10^8^ cells/mL. Consistently in this density range, the ratio *q*_lac_/*q*_glc_ was stable while the ratio *q*_amm_/*q*_gln_ increased above 1.6 × 10^8^ cells/mL, see Figures 6e,f.

From all these observations it was concluded that after the first transitory days, the cell metabolism was comparable at cell densities between 10^7^ and 2 × 10^8^ cells/mL except for a slightly modified glutaminolysis metabolism at density ≥1.6 × 10^8^ cell/mL. The large dataset gathered here at cell densities ≤35 × 10^6^ cells/mL indicated that the system, ATF or TFF, had no influence on the metabolism.

Furthermore, the representation of the growth rate in function of lactate or ammonium concentrations in Figures 5g,h indicated only random variations hence concentrations, up to 62 mM for lactate and 5.5 mM for ammonium, had no influence on the cell growth.

IS CHO CD XP medium supplemented with 3% IS-CHO Feed-CD XP was used in all the runs except in TFF#6 run on days 11–18 and in ATF#8 run on days 1–6, Hyclone PFCHO Liquid Soy medium supplemented with 3 % CHO Feed Bioreactor Supplement was also tested. No major difference in terms of cell density and viability was observed; showing that the results obtained here were not specific to the medium used.

### Filter and permeate pressures

#### Pressures During the TFF Perfusion Culture

**Figure 7:**
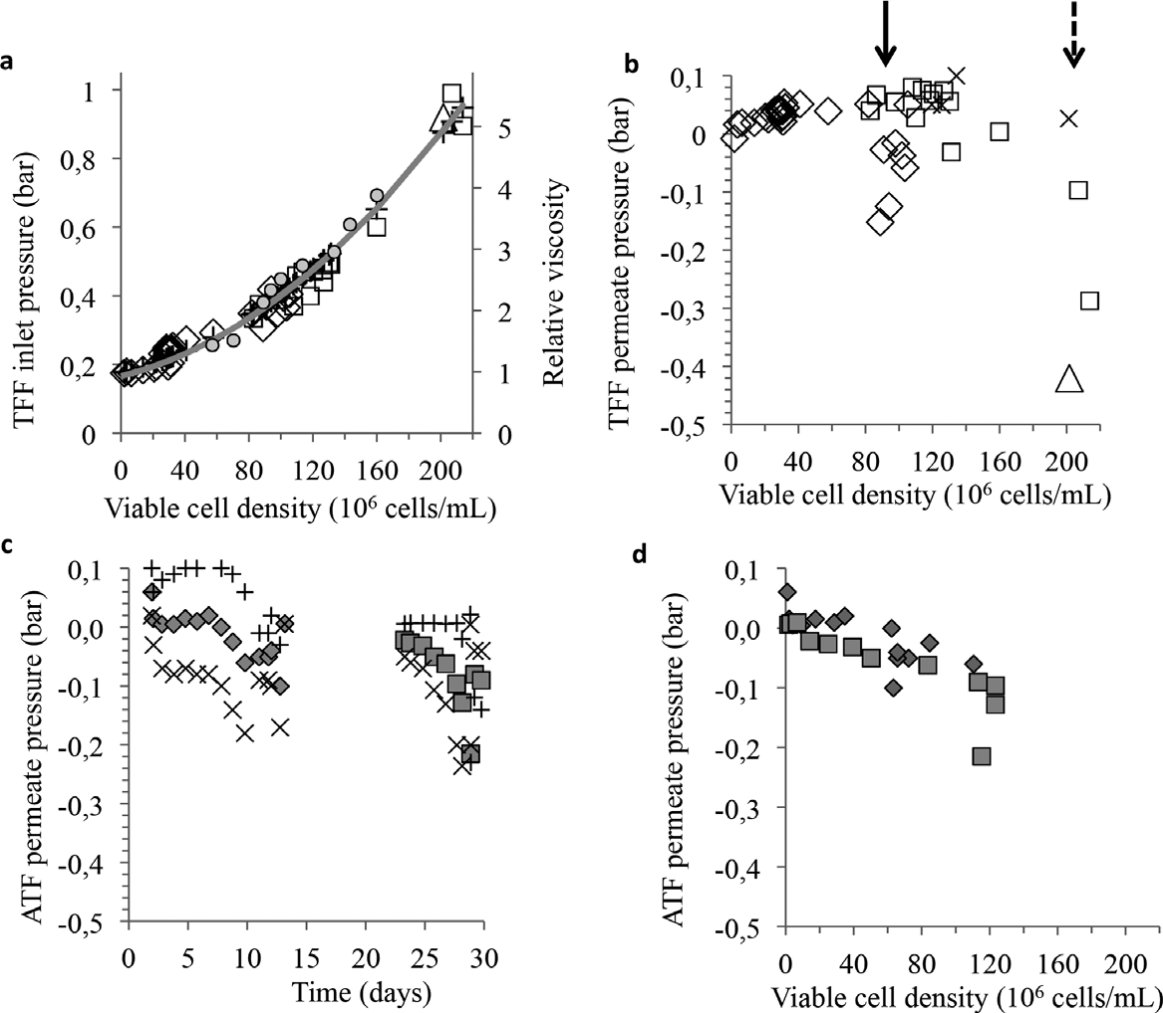
(a) *P*_1_ in TFF#10 run, using HF#1 (diamond), HF#2 (square), or HF#3 (triangle), *P*_1_est_ (+), modeled relative viscosity (continuous) and relative viscosity measured in concentrated cell broth cultivated in shake flasks (circle); (b) *P*_2_ in TFF#10 run, using HF#1 (diamond), HF#2 (square), HF#3 (triangle) or HF#2 and HF#3 in parallel (×). Vacuum observed when HF#1 membrane began fouling (continuous arrow) or when the cell density became extreme with *d*_i_ ≍ 0 μm (dashed arrow). (c) Average *P*_2_ in ATF#15A (diamond) and ATF#15B (square), *P*_2_ during pressure (+) and exhaust (×) cycles in function of time; (d) Average *P*_2_ in ATF#15A-B runs in function of cell density.

The inlet pressure (*P*_1_), the permeate pressure (*P*_2_), and the retentate pressure (*P*_3_) were monitored in TFF#10 run, see Figure 1. *P*_3_ was close to 0, expected value as it was the same as the bioreactor pressure (data not shown). One HF (HF#1) was used the first 30 days and changed for a new HF, HF#2, after occurrence of increasing vacuum of P_2_ for 6 days probably due to membrane partial fouling, see continuous arrow in Figure 7b. *P*_1_ was highly correlated to increasing cell density as can be seen in Figure 7a. As the perfusion rate was proportional to the cell density, *P*_1_ was also correlated with the perfusion rate however with a lower correlation (data not shown). At around 2 × 10^8^ cells/mL, *P*_1_ reached 0.8–1 bar hence a new HF (HF#3) was mounted. This however did not reduce *P*_1_ showing that the high pressure was not due to fouling. Then both the used HF#2 and the new HF#3 were connected to the bioreactor resulting in *P*_1_ decrease under 0.6 bar caused by a reduction of the hydrodynamic resistance from doubling the fiber number. This indicated that, at this cell density, a larger total lumen section area was necessary: by increasing the fiber number or increasing the lumen section. Notice however that increasing the filter area by prolonging the fiber length would not have helped since fouling was not responsible for the high pressure.

**Figure 8:**
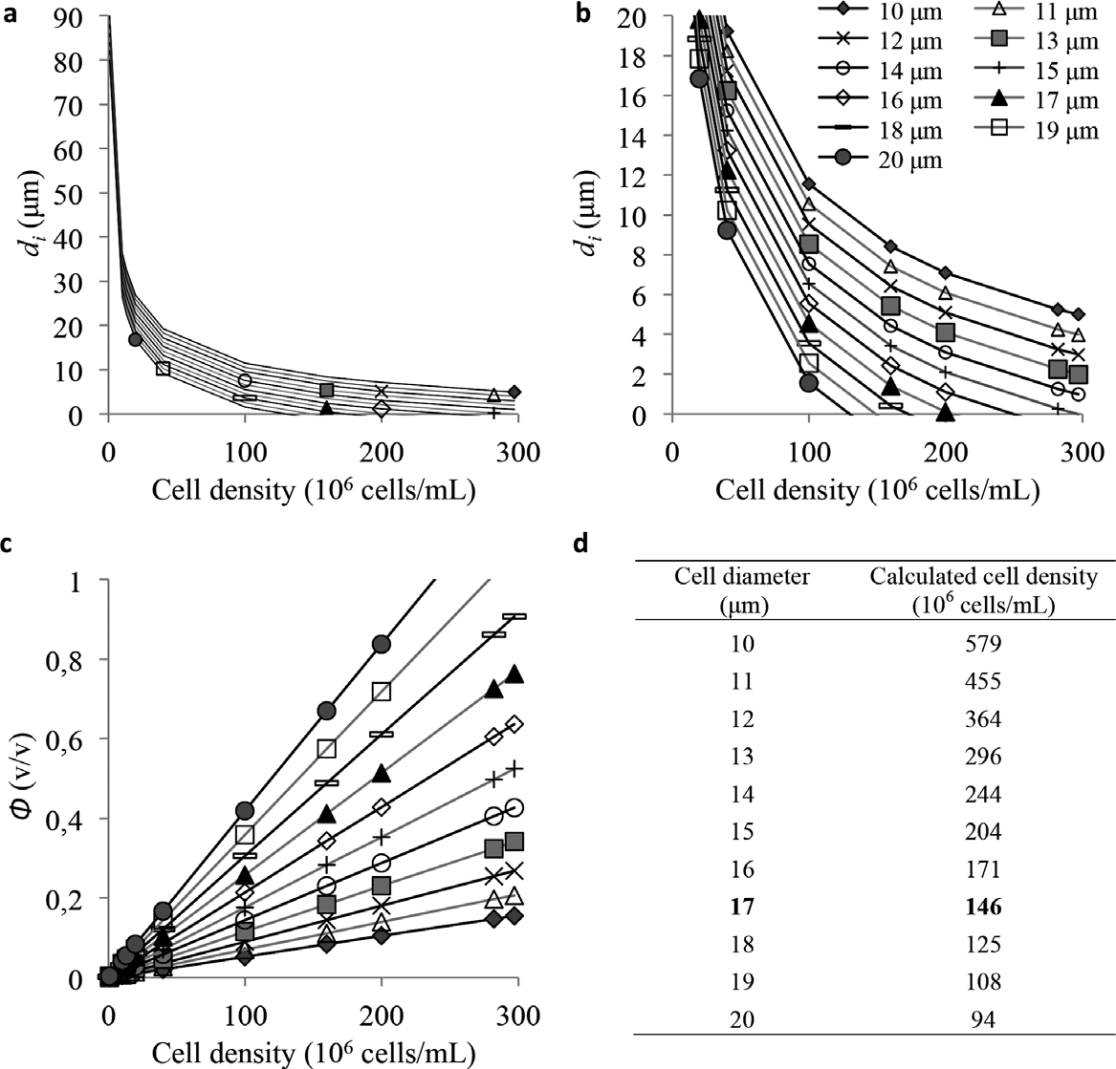
**(a)** Theoretical distance between the cells or intercellular distances, for uncompressible and spherical cells as a function of the cell density for different cell diameters; (b) zoom of (a) including legend for (a), (b), and (c); **(**c**)** fraction of solid (or cells) in the cell broth, *Φ*; (d) calculated cell densities for different cell diameters with 2 μm intercellular distance.

Except for the last days of HF#1 use (see above), *P*_2_ was close to 0 and slightly increasing with the cell density. However, at 2 × 10^8^ cells/mL a slight vacuum, −0.2 to −0.4 bar, was observed again, see dashed arrow in Figure 7b. After connection of both HF#2 and HF#3, no vacuum was observed anymore. The sudden *P*_2_ vacuum and higher *P*_1_ using only one HF were due to the high compactness of the cells and cell broth viscosity. It was calculated that for perfectly uncompressible spherical cells of 17 *μ*m *D*_cell_ (approximate *D*_cell_ average in this study), the theoretical distances between the cells or intercellular distances (*d*_i_),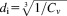 *− D*_cell_, were 12, 1.4, and 0 *μ*m at 4 × 10^7^, 1.6 × 10^8^, and 2 × 10^8^ cells/mL, respectively, see Figures 8a,b. Therefore, at 2 × 10^8^ cells/mL, the fluid properties became completely different: the cells were against each other while at lower cell density a liquid layer facilitating the movement of the cells relatively to each other, separated them. Thus when entering the narrower passage of the hollow fibers (total lumen section 0.39 cm^2^ vs. 0.71 cm^2^ dip tube inner diameter), this compactness caused a higher *P*_1_ due to a reduced movement of the cells with a reduced liquid layer. *P*_2_ vacuum was attributed to this compactness as the cells were against the inner fiber wall and probably obstructing the filter pores, as showed by the fact that *P*_2_ became slightly positive after connection of both HFs.

#### Cell Broth Viscosity

The cell broth relative viscosity, *η*_rel_, was estimated by a commonly used relationship, derived from theoretical development, for non-negligible solid fraction volume proposed by Thomas^43^ describing the viscosity of a mixture of liquid and solid spheres as a function of the fraction of spheres in suspension:


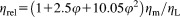
(6)

where *η*_m_ is the viscosity of the mixture of liquid and spheres, *η*_L_ is the viscosity of the pure liquid, *ϕ* is the volume fraction of spheres or solid in the mixture, that is, sphere volume × cell density. At high cell density, the solid fraction volume is not negligible anymore as it is at lower cell density, for example, *ϕ =* 0.1 and 0.5 at cell densities of 4 × 10^7^ and 2 × 10^8^ cells/mL, see Figure 8c. At low *ϕ*, the first two terms of eq. (6) describe well this relation but at higher *ϕ* the nonlinear term is important. Equation (6) model for the mixture relative viscosity was computed in Figure 7a. Data of the relative viscosity of concentrated cell broth cultivated in shake flask were added on Figure 7a indicative of the validity of this approach. The experimental data of viscosity found in the literature always present the pattern observed in Figure 7a independently of the particle nature as can be seen for concentrated rat aortic muscle CRL-1444 cells,^34^ yeast,^35^,^36^ plant cells,^37^ or oil.^38^ This pattern is the one predicted by the modeling theoretical approach.^43^ Empirical models in the literature have modified eq. (6) by adding higher power series in *ϕ* or exponential terms or modifying the quadratic term.

Interestingly, as can be seen in Figure 7a, *P*_1_ was proportional to the viscosity by a fitted factor *b* depending on the hydrodynamic resistance of the setting and the medium viscosity, *η*_L_. Hence a model of *P*_1_, *P*_1_est_, function of the viscosity was proposed; see eq. (7) and Figure 7a.


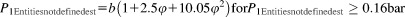
(7)

with *b*=0.175; *ϕ=Π D*_cell_*^3^ C_v_* 10^−12^*/6*; *D*_cell_ [*μ*m]; *C*_v_ [10^6^ cells/mL].

This relationship can be used to design the HF as described elsewhere (in preparation).

Above 1.5 × 10^8^ cells/mL and despite the slower growth rate, *D*_cell_ decreased to 16.4 *μ*m in average, value at which the theoretical *d*_i_ was 2 *μ*m. The implication of a reduction of *D*_cell_ is unknown hence it can be wise to keep the cell density below a value such that *D*_cell_ is unchanged and therefore *d*_i_≥2 *μ*m. The cell densities for *d*_i_ equal to 2 *μ*m were calculated for different *D*_cell_ in Figure 8d. At 17 *μ*m *D*_cell_, the highest theoretical cell density was 1.46 × 10^8^ cells/mL hence a maximal recommended cell density for the CHO cells used here.

#### Pressure During the ATF Perfusion Culture

*P*_1_ was not measured in this study to avoid invasive measurements in the dip tube between the bioreactor and the HF. Furthermore, the information about the pressures given by the ATF regulator could not be directly interpreted as the real pressure is depending on the orifice number. However, the information of *P*_1_ data observed in the TFF system could be used here to understand the above-mentioned ATF malfunction at 1.23 and 1.32 × 10^8^ cells/mL densities. At these cell densities in the TFF system, *P*_1_ was 0.52 and 0.54 bar. The ATF pump had to provide the same effect as observed in the TFF system: 0.53 bar for the exhaust and −0.53 bar for the vacuum cycle. With the settings used in this study, this vacuum value could not be reached and the ATF function was subsequently interrupted.

During the pressure cycle, that is, when the cell broth was pushed by the ATF pump into the bioreactor, *P*_2_ was ∼0 and mostly positive, see Figure 7c. During the exhaust cycle, that is, when the cell broth was pulled by vacuum from the bioreactor, *P*_2_ was negative and with higher absolute amplitude than in the pressure cycle. The resulting profile of *P*_2_ average was completely different for the ATF compared to the TFF system: it decreased with the cell density and *D* instead of being constant or slightly increasing, see Figures 7b,d. *P*_2_ was positive in the TTF system and the ATF system during the pressure cycle when the upstream pressure was positive while it was negative when the upstream pressure was negative.

#### Indicative HF Capacity

In run TFF#10, one HF lasted 30 days, including 1 week at ∼10^8^ cells/mL, and another one 14 days (at 1.1 × 10^8^ cells/mL for 11 days followed by increase to 2.1 × 10^8^ cells/mL). In TFF#6 and ATF#9 runs, one HF was used during 22 and 27 days, respectively. Potentially two factors could explain this long lasting HF despite very high cell densities: (i) the use of 1 L/min in the recirculation loop allowing to generate shear rate at the filter surface, preventing filter fouling as observed in a study of Russotti et al.^39^ for yeast harvest by microfiltration and as mentioned in Castilho and Medronho^40^; and (ii) the very high cell viability from healthy growing cells resulting in reduced cell debris and DNA accumulation. A very high cell concentration of 1.32 × 10^8^ cells/mL was obtained using one HF cartridge in ATF operation. It should be emphasized that the present data were not sufficient to draw general conclusion of the HF usage length with TFF or ATF systems.

### DO control, bubble occurrence in the dip tube, and shear rate

DO was controlled by varying the rocking rate between selected maximal and minimal values (MAX-min) with controlled rate step change (Step). The O_2_ percentage in the inlet gas was increased to 100% O_2_ from cell densities ≥0.8 × 10^8^ cells/mL; giving the limitation of DO control by varying the O_2_ inlet percent. It was identified that the optimal settings were either a difference MAX-min of 4 rpm with 2 rpm Step or a difference MAX-min of 3 rpm with 1 rpm Step, for example, MAX-min of 26-22 rpm and 2 rpm Step at cell density 20–35 × 10^6^ cells/mL, see Table 2. This DO control provided a SP tracking between SP±5% and SP±15% without negative impact on the cell growth or viability. It was also key for ATF operation to avoid air accumulation in the dip tube: using MAX-min of 26-22 rpm resulted in alternated periods of a few minutes at 26 rpm, where bubble(s) occurred in the dip tube, and a few minutes at 22 rpm, where bubbles were pushed away from the dip tube. Applying MAX-min of 28-24 rpm did not allow bubble removal from the dip tube and a viability decrease was observed, for example, on day 10 in ATF#9 run, while this had no influence using the TFF system.

A concern during the ATF operation was the occurrence and sometimes accumulation of gas bubbles in the dip tube and/or the HF. When bubbles entered in the dip tube, they had a tendency to stay due to the tube arch-shape, see Figures 1a,b. Hence increasing the recirculation flow rate improved the bubble removal from the dip tube by chasing them back into the bioreactor. The recirculation rate was set to 1 L/min. During ATF#9 run, a recirculation rate of 0.7 L/min was tested as this latter value corresponded to the rate applied at larger scale. Above 20 × 10^6^ cells/mL, a higher rocking was necessary for the DO control generating more bubbles. It was observed that a 0.7 L/min recirculation flow rate was not sufficient to remove the bubbles entrapped in the dip tube/HF and that this resulted in viability decreasing from 97% to 93.5% at days 8 and 9. Therefore, from day 9 and onward, the flow rate was increased to 1 L/min (3,400 s^−1^ *γ*) allowing to maintain the viability at this latter level instead of further decreasing. Increasing the recirculation rate from 0.7 to 1 L/min provoked the release of trapped gas bubble from the dip tube and HF system, which were visually observed.

The volume of the TFF recirculation loop was minimized to 250 mL to reduce the cell exposure to a noncontrolled environment outside the bioreactor. The recirculation was 0.3 L/min (1,000 s^−1^ *γ*) in TFF#6 run and 0.7 L/min (2,400 s^−1^ *γ*) from days 1 to 8 in TFF#10 run, resulting in a residence time <1 min. From day 9, a recirculation rate of 1 L/min was applied to mimic the shear rate in the ATF system. No impact of these shear rates was observed on CHO cell growth, viability, and metabolism. These results are in agreement with Castilho and Medronho^40^ showing that myeloma, hybridoma, and SF-9 cells were not damaged at average shear rates up to 3,000 s^−1^ using a TFF system. On the contrary, in Zhang et al.,^41^ a 1,266 s^−1^ shear rate using a mixed cellulose ester HF affected hybridoma cell growth. A cell density ∼30 × 10^6^ cells/mL was maintained for 14 days in TFF#6, ATF#9, and TFF#10 with one HF without requiring a washing line as described in Kawahara et al.^42^

## Conclusions

Two perfusion processes in wave-induced bioreactor using ATF or TFF systems for cell separation were successfully developed. The bioreactors and filter cartridges were identical for both systems differing only for the cell separation device. The systems were developed and studied at densities maintained between 20 and 35 × 10^6^ cells/mL in several runs where consistency was also demonstrated. The perfusion was based on applying a cell specific perfusion rate of ∼0.06 nL/cell/day between 0.2 and 2.14 × 10^8^ cells/mL and supplementing glucose and glutamine separately.

A very high cell density was maintained by daily cell bleeds around 0.9–1.3 × 10^8^ cells/mL in growing phase at high viability using the TFF system, at perfusion rate between 4.5 and 5 RV/day. This process was maintained during more than 2 weeks showing its consistency before the cell density was further increased to test the system limit. With the present settings, a maximal cell density of 2.14 × 10^8^ cells/mL was reached. To our knowledge, it is the first time that a CHO cell density of more than 2 × 10^8^ cells/mL was achieved in a wave-induced bioreactor.

Using ATF, the maximal cell density was 1.3 × 10^8^ cells/mL. It was limited by the vacuum capacity failing to pull the highly viscous fluid for the present settings. To overcome this, several possibilities could have been applied: for example, (1) the vacuum effect should have been lower than −0.53 bar, (2) the bioreactor should have been pressurisable, which was not possible for a disposable bioreactor, (3) the total lumen section area should have been increased by increasing the number of fibres or increasing the lumen section.

Using TFF, the cell density was limited by the membrane capacity for the encountered high viscosity and the *p*CO_2_ level. A higher cell density would have required a larger total lumen section area and an improved CO_2_ removal. It was also calculated that at 2 × 10^8^ cells/mL, the theoretical intercellular distance was zero for the present cell line, potentially leading to a more drastic modification of the fluid properties. Furthermore, the cell diameter decreased above 1.5 × 10^8^ cells/mL density. The consequences of this reduction are unknown even though the cell continued to grow. The cell metabolism was independent of the system used, ATF or TFF, and was comparable at densities 10^7^ to 1.6 × 10^8^ cells/mL, above which the glutamine metabolism became different. A conservative conclusion was then that a cell density ≤1.46 × 10^8^ cells/mL would be preferred for the present cell line. It would allow to keep a metabolism comparable to lower cell density and to maintain a recommended theoretical intercellular distance≥2 *μ*m. The difference between this theoretically recommended cell density limit of 1.46 × 10^6^ cells/mL and the maximal density obtained here, 2.1 × 10^8^ cells/mL, was quite large ensuring a large safety margin.

With the present settings, the TFF system allowed reaching higher cell densities. It was also more robust against the presence of bubbles. In particular, using the ATF system, the agitation had to be reduced to avoid gas bubble accumulation in the dip tube between the bioreactor and the HF since this was susceptible to jeopardize the filtering function. This put higher constraint on the oxygenation control and prevented the application of increased agitation to remove excessive CO_2_. The working volume was important for the foam formation and the presence of bubbles in the dip tube: at identical agitation, smaller volumes resulted in higher bubble accumulation. 4–5 L working volume was recommended when targeting very high cell densities necessitating high agitation. AF was added to prevent foam formation but even in absence of foam on the liquid surface, gas bubbles were present in the liquid phase and were dragged into the dip tube by the fluid motion. This was an issue for the ATF system, which was addressed by limiting the bioreactor rocking and by using alternating faster and slower rocking speed in the DO control strategy. The presence of bubble had no effect in the TFF system during our study; however, it was observed that the bubble amount increased with increasing rocking rate. Therefore, milder rocking rate ranges were preferred for the DO control as bubbles entering the HF would jeopardize the filter function even in the TFF system. From these different points of view, the TFF system was more advantageous than the ATF system for the production of cells. On another hand, as presented in Part II, the product of interest was retained by the HF, and this retention was higher for the TFF than for the ATF system.

Pushing the cell density to very high values in the range encountered in tissue led to several interesting pieces of information: For the present cell line, the high cell density by itself did not affect the culture until a cell density of 1.5 × 10^8^ cells/mL, value at which the theoretical intercellular distance was 2 *μ*m. Above this value, the cell diameter decreased and slightly above this value, the glutamine metabolism began to diverge compared to lower cell densities. Most of the observations about the perfusion process made in the present article are probably applicable to perfusion performed in stirred tank.

As expected, the cell broth viscosity increased with the cell density and had a significant impact on the process. In today's industrial processes, a cell density considered as middle-high or high is 20 to 50 × 10^6^ cells/mL. In these processes, the viscosity is slightly above the medium viscosity and has only a limited influence on the process. In our study at very high cell densities, the viscosity had a visible impact on the filtration by HF, leading to a high pressure in the system and giving quantitative information about the present setting limitation useful for the HF design and dimensioning.
